# Early new bone formation in ovine intramuscular defects: comparison between different silicate-containing calcium phosphate synthetic bone grafts

**DOI:** 10.1186/s13018-025-05740-0

**Published:** 2025-04-11

**Authors:** Iain R. Gibson, Viviana R. Lopes, Rema Oliver, Tian Wang, Dan Wills, Tom Buckland, William R. Walsh

**Affiliations:** 1 Institute of Medical Sciences, School of Medicine, Medical Sciences and Nutrition, University of Aberdeen, Foresterhill, Aberdeen, AB25 2ZD Scotland, UK; 2 Department of Chemistry, University of Aberdeen, Meston Walk, Aberdeen, AB24 3UE Scotland, UK; 3 OssDsign AB, Rapsgatan 23 A, SE 754 50 Uppsala, Sweden; 4 Surgical and Orthopaedic Research Laboratories, Prince of Wales Clinical School, UNSW Sydney, Sydney, NSW Australia

**Keywords:** Synthetic bone graft, Osteoinduction, Silicate, Apatites, Nanoscale, Bone formation, Intramuscular defect

## Abstract

**Background:**

The property of in vivo osteoinductivity (OI) has been reported in synthetic calcium phosphates bone grafts, including silicate-containing calcium phosphates in different animal intramuscular defect models. However, most studies that have evaluated osteoinductivity in these models only focus on the formation of new bone at only one endpoint, typically 12 weeks, and without reporting evidence of graft resorption.

**Methods:**

Three clinically available silicate-containing calcium phosphate bone graft substitutes were characterised and then implanted into an ovine intramuscular defect model for 6 or 12 weeks to assess their bone forming potential. Bone formation was evaluated with radiographs, micro-CT (µCT) and qualitative histology.

**Result:**

The main physical/chemical differences between the three materials were the morphology and surface areas of the graft materials, but also the form that the silicate was incorporated. One of the bone grafts with a nano-scale microstructure (nano-Si-Ap) resulted in significant new bone formation that was sufficient to bridge between granules after 6 weeks which progressed further after 12 weeks implantation, and evidence of graft resorption/remodelling was observed at both time points. The other nano-scale bone graft (nano-SiO_2_-HA) showed a more limited quantity of new bone formation at 6 and 12 weeks and did not show evidence of resorption/remodelling. The bone graft with a micron-scale microstructure and low surface area (micro-Si-CaP) exhibited very limited evidence of new bone formation or resorption at either time points.

**Conclusions:**

Implantation in ovine intramuscular defects was found to be an effective model to differentiate the relative bone forming potential of three silicate calcium phosphate bone grafts, particularly using a short implantation time of only 6 weeks. Positive outcomes in such a pre-clinical model when evaluating synthetic bone graft substitutes may be clinically relevant to their potential use in challenging bone defects.

**Supplementary Information:**

The online version contains supplementary material available at 10.1186/s13018-025-05740-0.

## Background

The most significant development towards improving the bioactivity of synthetic CaP ceramic materials as bone graft substitutes over the last two decades has been to make them not only osteoconductive but also osteoinductive [1]. In order to be osteoinductive, synthetic CaP materials need to meet certain specifications in terms of macrostructure, microstructure and chemical composition although the osteoinduction pathway is still unknown and is likely complex and multi-factorial [2]. An osteoinductive response has been associated with the development of CaP materials with an optimised curvature of macroporous structures [3], the presence of $B$-tricalcium phosphate ($B$-TCP) in addition to hydroxyapatite (HA) [4], increased levels of strut microporosity in macroporous ceramics [5], submicron-scale ceramic structures [6], and modification of the surface microstructure [7]. More recently, the incorporation of silicon or silicate into synthetic CaPs has been shown to contribute towards an osteoinductive response in vivo [5, 8, 9]. The material from one of these studies was also composed of hydroxyapatite nano-scale crystals [8]. Nanoscale CaP materials, with a primary particle size dimension of 50–100 nm, bear a higher specific surface compared to traditional ceramic CaPs that have a primary particle size-scale of 0.3–5 μm, which can translate to an improved ability to engage the host’s immune and progenitor cells at the nanoscale, resulting in enhanced outcomes [10].

There are few materials that are clinically available that combine the concepts of silicate ion incorporation and nanoscale surface structure in a single bone graft. In a recent preclinical study using posterolateral fusion in rabbits, a nanosynthetic graft material, used as an extender to autograft, showed a similar progression to fusion to the autograft control between 6 and 12 weeks, with 100% fusion observed at a 26-week time point [11]. This graft material (described as Osteo^3^ ZP Putty, but marketed as OssDsign Catalyst) was a novel silicate calcium phosphate composed of crystals that are comparable in size scale to bone mineral crystals. In contrast to the biphasic calcium phosphate ceramic control group (Mastergraft Putty), new bone formation was observed at the centre of the fusion mass, far from the transverse processes, after only 6 weeks, similar to the autograft control group, with histological evidence of bone formation by both endochondral and intramembranous ossification.

Successful fusion of the spine requires new bone formation in two different environments: close to host bone and at the centre of a fusion mass at a distance from host bone. To test this a preclinical intramuscular defect model that aims to recapitulate the challenging environment of the centre of a fusion mass, away from local bone, was utilised to better understand the bone formation observed in the rabbit PLF study. A common theme in the various large animal models that have been utilised to study the osteoinductivity of CaP materials is the timepoints reported as end-points to assess osteoinductivity, with the earliest time point in the vast majority of studies as 12 weeks [21], which is notably later than the initial end-points in other pre-clinical models for assessing bone formation with CaPs, which is typically 4–6 weeks. Large animal models provide the opportunity for multiple defects per animal, and the implantation of quantities of bone graft that are more clinically relevant, compared to small animal models of osteoinduction.

This paper describes the presence or absence of bone formation when graft material was implanted into defects created in the paraspinal muscle of sheep at 6 and 12 weeks. A nanosynthetic silicate calcium phosphate bone graft was compared to two other silicate calcium phosphate bone grafts that have been reported as being osteoinductive in pre-clinical studies, and which all utilise the same aqueous hydrogel carrier, in this pre-clinical OI model. These three bone grafts had different amounts of silicate ions, but also different surface micro- and/or nanostructures.

## Materials and methods

### Graft materials

Three different commercial bone graft substitutes were used in this study: (1) a nanoscale silicate-substituted apatite consisting of granules in a poloxamer carrier (nano-Si-Ap; OssDsign Catalyst, OssDsign); (2) a nanocrystalline hydroxyapatite embedded in a silica-gel (SiO_2_) matrix consisting of granules in a poloxamer carrier (nano-SiO_2_-HA; Nanobone SBX Putty, Artoss); (3) a micron-scale silicate-substituted calcium phosphate consisting of granules in a poloxamer carrier (micro-Si-CaP; Inductigraft, or Altapore in the USA, Baxter). For the implantation study all bone grafts were used directly from the sterile packaging.

For physical characterisation of the bone graft materials the poloxamer carrier was removed by soaking the graft materials in cold water (4–5 °C), at 1.3 g putty per 100 ml of water, until all granules were released and dispersed in the water, with gentle mixing of the mixture for approximately 5 s every 5 min. Before adding to water, the volume of putty used was weighed, giving the total mass of granules and poloxamer carrier. After multiple washes (4–5 times with deionised water), granules were collected by filtration and then the collected granules were dried overnight at 80 °C, weighed, then stored until analysed. For XRD and FTIR analysis the granules were ground to a fine powder in a mortar and pestle.

### Characterisation methods

#### X-ray diffraction (XRD)

X-ray diffraction data of powders were collected between 10 and 80° 2θ, using a step size of 0.0263°/step and a scan time of 450 s/step, using a Panalytical Empyrean X-ray diffractometer (Malvern Panalytical, UK) operating at 40 kV and 40 mA, equipped with a primary monochromator resulting in Cu K_α1_ X-rays of wavelength 1.54056 Å. Confirmation of the phase composition was carried out using HighScore (Malvern Panalytical, UK), using the ICDD reference PDF no. 96–900- 215. The crystallite sizes of samples were calculated using the Scherrer equation using the (002) diffraction peak. A silicon standard was used as an internal calibration to account for instrument broadening.

#### Fourier transform infra-red (FTIR) spectroscopy

FTIR spectra of the powdered samples were obtained using a Diamond/ZnSe ATR cell attached to a Spectrum Two™ spectrometer (Perkin-Elmer, UK). Absorbance spectra were collected between 4000 and 400 cm^−1^ at a resolution of 2 cm^−1^, averaging 32 scans.

#### Scanning electron microscopy (SEM)

Scanning electron microscopy (SEM) images of the granules were collected using an EVO MA 10 SEM (Zeiss, Germany), using an accelerating voltage of 10 kV. Granules were fixed to aluminium stubs using adhesive carbon tape and conductive paint and were then coated with Pd/Au. Average granule sizes were determined by measuring the longest dimension of > 35 granules from images collected at 40 × magnification using Image J [12].

#### Surface area measurement

The surface area of granules was determined from nitrogen adsorption isotherms that were collected using a Micromeritics TriStar 3000 gas adsorption analyzer (Micromeritics Instrument Corp., USA). The specific surface area, SSA (m^2^/g), of granules was determined from the collected data using the BET method (MicroActive software, Micromeritics Instrument Corp. 2012). Granules were degassed under flowing nitrogen gas at 250 °C for 4–6 h prior to the measurement of gas adsorption.

### In vivo implantation study

#### Animal model and surgical procedure

The in vivo study was approved by the local Animal Care and Ethics Committee at the (Approval 18/115 A) at the UNSW, Australia. A total of 6 skeletally mature adult (> 24 months) male Border Leicester Merino Cross sheep were used in this study. Two implantation time points were studied, with a distribution of 4 animals at 6 weeks and 2 animals at 12 weeks.

The surgical procedure used was reported in a previous study [13]. Briefly, the surgical sites were in the left and right paraspinal muscles; this model allows two rows of four implantation sites in each muscle, providing up to 16 separate sites per animal. Implantation sites were created with dissecting scissors placed into the muscle and then opening in two planes. An open bore syringe containing 1cc of graft material was placed into the defect adjacent to the scissors, then the scissor was removed, and the graft material was extruded from the syringe into the defect site. The defect site was then closed with a suture. Graft materials were assigned randomly to the different implantation sites.

The following groups were used for assessing the impact of different silicate amounts and nano/microstructure on OI bone formation:

1. nano-Si-Ap (OssDsign Catalyst, OssDsign)
2. nano-SiO_2_-HA (Nanobone SBX Putty, Artoss)
3. micro-Si-CaP (Inductigraft, or Altapore in the USA, Baxter).

All the sheep were healthy and all the surgical implant sites healed well without any significant wound complication. After 6 and 12 weeks of implantation, sheep were sacrificed and the right and left paraspinal muscles containing the bone grafts with surrounding tissues were retrieved and the implant sites harvested, X-ray and micro-CT analysis was performed, and samples were then processed for histological analysis. No visual signs of inflammation or adverse host tissue reaction were observed at harvest.

### Histology

Explants were decalcified in 10% formic acid in phosphate buffered formalin at room temperature for 3–4 days and then each explant was cut into two pieces. These were embedded in paraffin and sectioned (5 microns) using a Leica Microtome (Leica Microsystems Pty Ltd, North Ryde, Australia). Sections were stained with either haematoxylin and eosin (H&E) or Tetrachrome and examined to qualitatively examine local cell and tissue responses using an Olympus light microscope (Olympus, Japan) with a DP72 high-resolution video camera (Olympus, Japan). Sections were graded by 3 trained observers blinded to group and time point for evidence of osteoinductivity based on the percentage of new bone formation from 0 (no new bone), 1 = 1–25%, 2 = 26–50%, 3 = 51–75%, 4 = > 75%). Data were analysed using non-parametric Kruskal Wallis test and significance values were adjusted by the Bonferroni correction for multiple tests using IBM SPSS Ver 27.

### Radiography–X-rays and uCT

Radiographs of harvested muscles were obtained in the anteroposterior (AP) plane using a Faxitron and digital plates (AGFA CR MD4.0 Cassette). The digital images were processed using an AGFA Digital Developer and workstation (AGFA CR 75.0 Digitiser Musica, AGFA, Germany); the DICOM data were converted to JPEG images using ezDICOM medical viewer.

Micro computed tomography (μCT) scanning (approximate scan thickness of 50 microns) was performed as previously described [14] on all implantation sites using an Inveon in-vivo micro computed tomography scanner (Siemens Medical, PA, USA).

## Results

### Granule preparation for chemical and physical characterisation

**Fig. 1:**
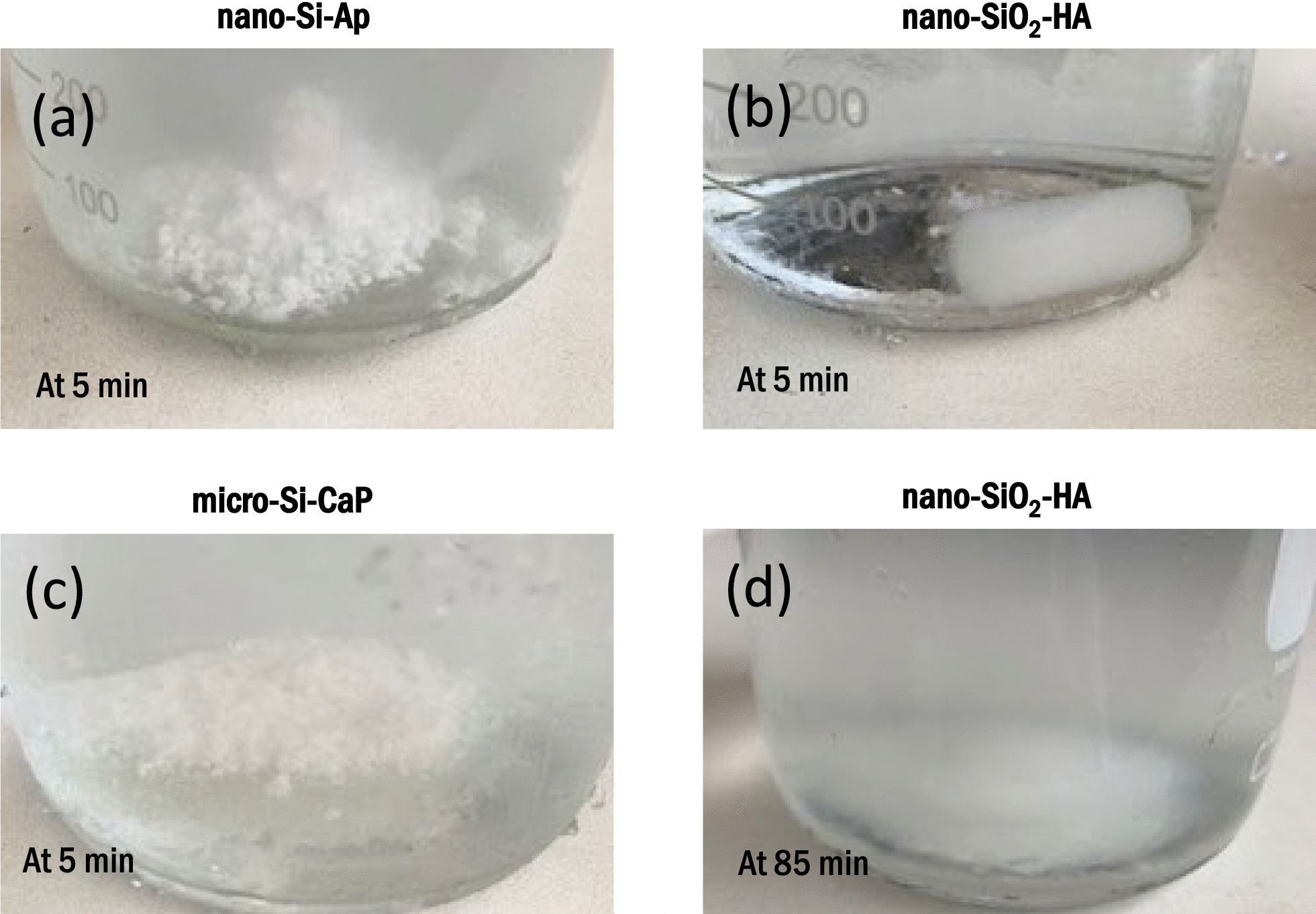
Photographs of the putty samples after immersing in cold water for 5 min. **a** nano-Si-Ap, **b** nano-SiO_2_-HA and **c** micro-Si-CaP, and after 85 min, **d** nano-SiO_2_-HA

The granules could be removed effectively from the poloxamer carrier by immersion in cold water, but the time required to achieve this varied between samples. The nano-Si-Ap and micro-Si-CaP granules were dispersed from the poloxamer after 5 min (Figs. 1a and c, respectively), whereas the nano-SiO_2_-HA sample showed no break-down of the putty after 5 min (Fig. 1b) and required 85 min to achieve complete dispersion (Fig. 1d).

The mass ratio of granules to aqueous poloxamer carrier were determined by the mass difference between the mass of the original putty and the mass of the dried retrieved granules, with the results listed in Table 1. Although the time required to remove the poloxamer carrier in cold water was much greater for nano-SiO_2_-HA compared to the other samples, the percentage mass of granules in the nano-Si-Ap and nano-SiO_2_-HA putties were comparable, whereas the micro-Si-CaP Putty had a significantly larger quantity of granules per cc of putty. The nano-SiO_2_-HA extruded from the delivery syringe was considerably stiffer than the other two putty materials and could not be easily moulded by hand, whereas the nano-Si-Ap and micro-Si-CaP putties could be very easily shaped and moulded by hand after extrusion from their delivery syringes.

**Table 1:** Various physical parameters of the granules of the three putty samples studied

	nano-Si-Ap	nano-SiO_2_-HA	micro-Si-CaP
*Putty composition*^*a*^			
wt% of granules	30%	32%	41%
wt% of poloxamer gel carrier	70%	68%	59%
Mean granule size (mm)^b^	2.8	1.3	2.1
Crystallite size (nm)^c^	29.9	22.7	N/A*
Surface area (m^2^/g)^d^	26	186	0.2^#^

^a^determined by mass loss after removal of poloxamer carrier in ice cold water

^b^determined from SEM images

^c^determined from XRD data

^d^determined from BET method from nitrogen adsorption measurements

^*^peak width was too narrow to determine a crystallite size

^#^surface area could only be determined by single point determination at 0.1994

### Granule characterisation

**Fig. 2:**
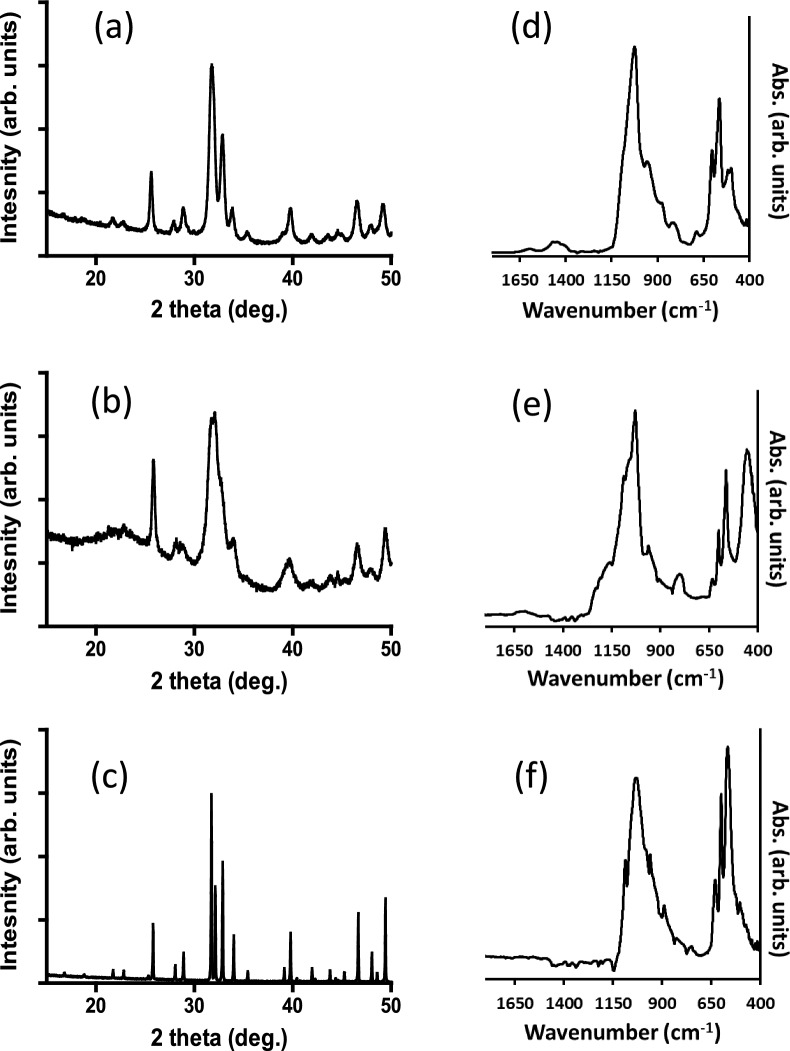
X-ray diffraction patterns (**a**–**c**) and FTIR spectra (**d**–**f**) of the granules removed from the putty samples of (**a**, **d**) nano-Si-Ap, (**b**, **e**) nano-SiO_2_-HA and (**c**, **f**) micro-Si-CaP

X-ray diffraction patterns and FTIR spectra of the powdered granules that were removed from the putty samples are shown in Fig. 2. All three samples exhibited diffraction peaks that corresponded to a hydroxyapatite-like structure, referenced to the ICDD standard for hydroxyapatite (PDF file no. 96–900 - 215), with no identifiable impurity phases(s). There were significant differences, however, in the diffraction peak shapes which reflects differences in the crystallite sizes of the samples. The nano-Si-Ap and nano-SiO_2_-HA samples resulted in broad diffraction peaks, although the peaks from the former, Fig. 2a, were better resolved than the latter Fig. 2b. In contrast, the micro-Si-CaP sample produced very sharp and narrow diffraction peaks, Fig. 2c, typical of a very crystalline material.

An estimation of the crystallite sizes of sub-micron sized materials can be obtained from peak broadening in the X-ray diffraction patterns using the Scherrer equation. Values for nano-Si-Ap and nano-SiO_2_-HA samples are shown in Table 1 and the crystallite sizes were comparable.

The FTIR spectra of the powdered granules all showed the main characteristics of a calcium phosphate apatite, with phosphate vibrations at approximately 1000–1100, 960 and 450–630 cm^−1^ [15], Fig. 2d–f. The spectra of nano-Si-Ap samples and micro-Si-CaP samples, Figs. 2d and f, also showed vibrations corresponding to silicate vibrations, consistent with previous studied of silicate-substituted calcium phosphates, at approximately 890 and at 510–540 cm^−1^ [16]. A strong vibration at approximately 630 cm^−1^ was observed in spectra for both nano-SiO_2_-HA samples and micro-Si-CaP samples, Fig. 2e and f, which relates to the hydroxyl group in hydroxyapatite [15]. The spectrum of the nano-SiO_2_-HA sample was consistent with the manufacturer’s description of the material, with vibrations corresponding to a hydroxyapatite phase and of a SiO_2_ matrix, with broad vibrations at approximately 800 and 440 cm^−1^ consistent with silica gel matrix [17].

The general morphologies of the granules of the three bone graft materials are observed in the low magnification SEM images in Figures S1 (supplementary data). The micro-Si-CaP consisted of irregular shaped granules that contained large levels of microporosity. Granules of nano-Si-Ap and nano-SiO_2_-HA granules were comparable in appearance, but the former were larger than the latter; this was confirmed by measurement of the longest dimension of a large number of granules, with the mean sizes reported in Table 1. Higher magnification images provide an indication of the ceramic microstructure of the micro-Si-CaP sample, with the presence of microporosity within the ceramic microstructure, Figure S1F. The nanoscale dimensions of the primary particles of the nano-Si-Ap and nano-SiO_2_-HA granules made imaging challenging by SEM, with the higher magnification images not providing clear imaging of these nanoparticles, Figure S1D and S1E; high resolution FEG-SEM of the nano-Si-Ap nano-scale morphology has been reported previously [11].

The different microstructures and morphologies of the primary particles of the three materials resulted in significant differences in the specific surface areas (SSA) of the granules, Table 1. Values for the nano-Si-Ap and nano-SiO_2_-HA granules could be determined using the BET method and showed high values of SSA of 26 and 186 m^2^/g, respectively. The crystalline ceramic nature of the micro-Si-CaP sample was associated with a very low value of SSA of only 0.2 m^2^/g, although this could only be determined from a single point determination from the BET method.

### Evaluation of the tissue response to implanted materials

**Fig. 3:**
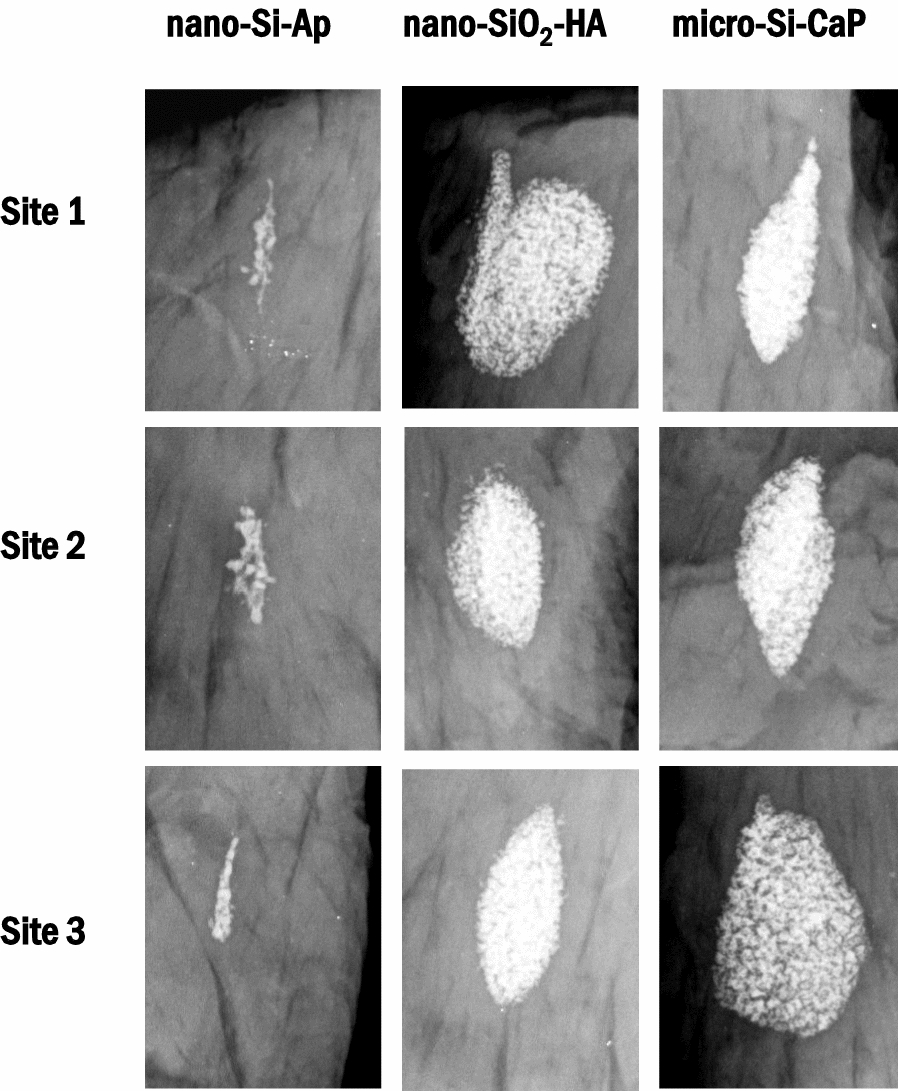
Representative high-definition Faxitron radiographs at three different sites of nano-Si-Ap, nano-SiO_2_-HA and micro-Si-CaP after 12 weeks implantation in the paraspinal muscle of sheep, respectively. Each muscle site was implanted with 1 cc of each material

All harvested samples were radiographed, and no evidence of adverse reactions was noted. There was evidence of graft resorption for nano-Si-Ap group when compared to the other two groups, Fig. 3. The radiographs confirmed the materials had yet to completely resorb at 12 weeks. However, it was not possible to distinguish the implanted material from any newly bone formed at any time points using this method.

**Fig. 4:**
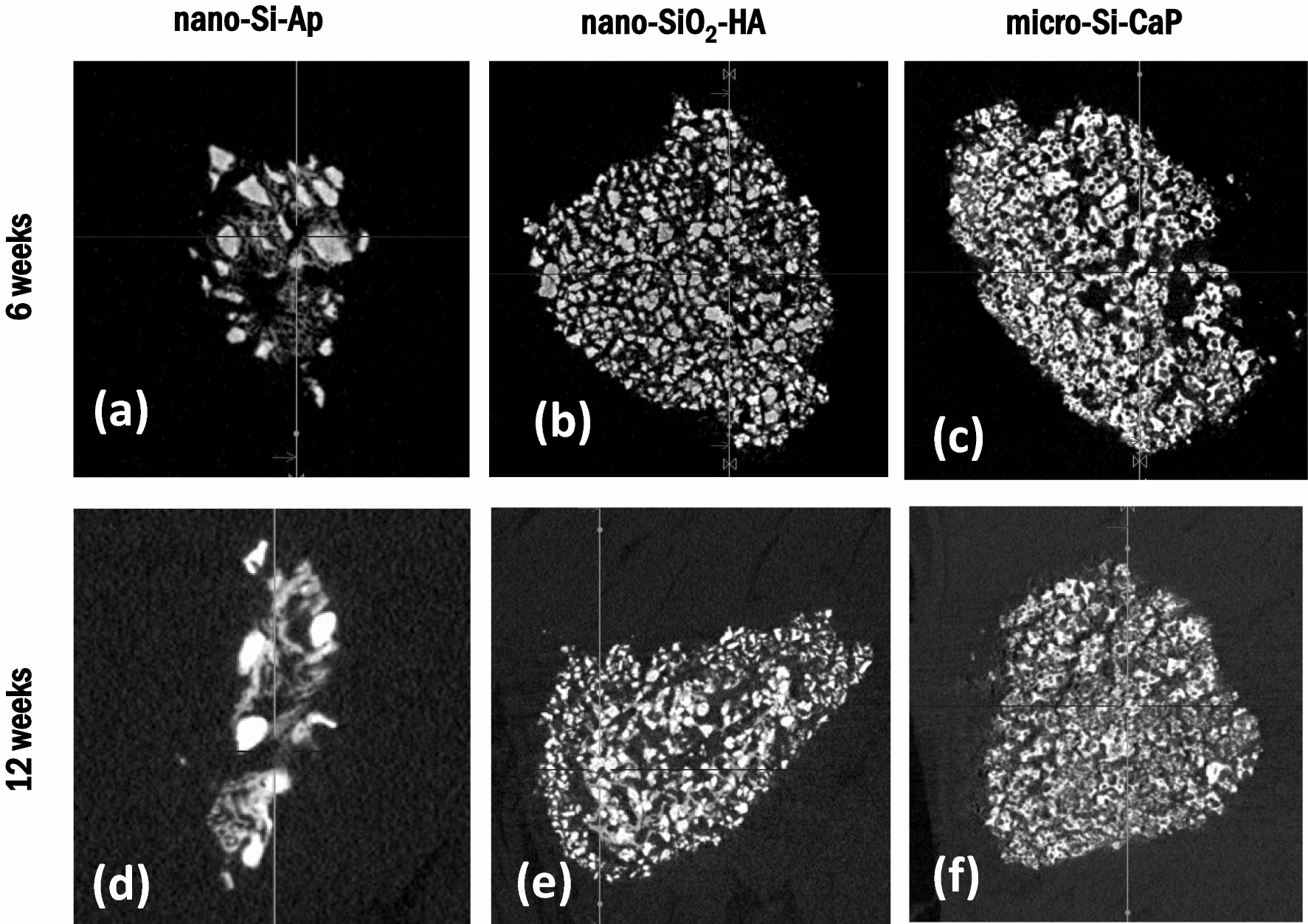
2D representation of micro-CT sections of (**a**, **d**) nano-Si-Ap, (**b**, **e**) nano-SiO_2_-HA and (c,f) micro-Si-CaP after 6 (**a**–**c**) and 12 (**d**–**f**) weeks implantation in the paraspinal muscle of sheep, respectively

Representative 2D images from the micro-CT analysis of the explants showed new bone formation after 6 weeks implantation in the nano-Si-Ap group, Fig. 4a and d after 6 and 12 weeks, respectively. Abundant trabecular-like mineralisation could be observed bridging between the granules for nano-Si-Ap, and similar structures were not observed in the nano-SiO_2_-HA or micro-Si-CaP groups, Fig. 4b and e, c and f respectively.

**Fig. 5:**
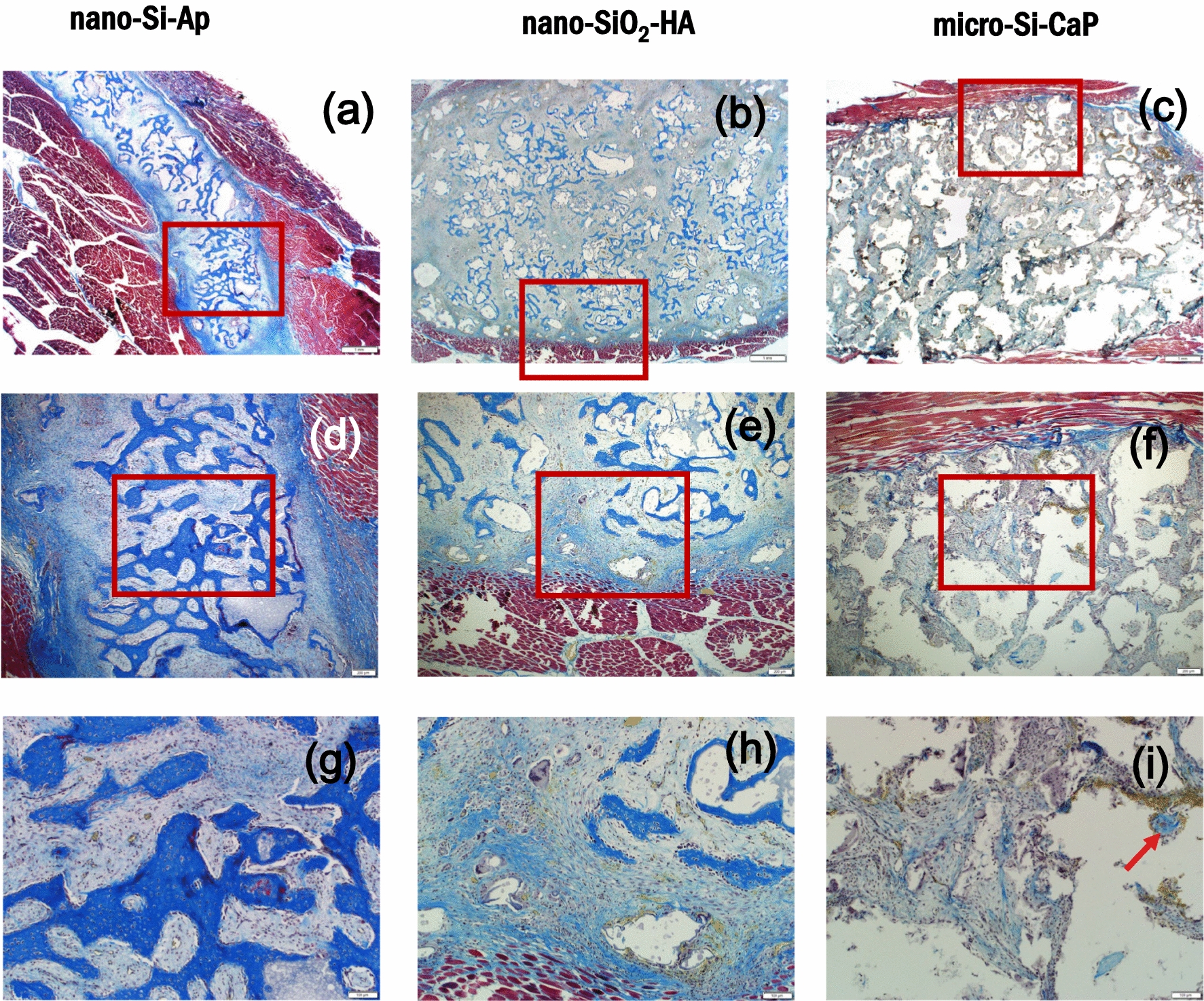
Histological sections with tetrachrome staining of the putty samples implanted in muscle defects for 6 weeks for (**a**) nano-Si-Ap, (**b**) nano-SiO_2_-HA and (**c**) micro-Si-CaP and at medium magnification (d,e,f, in the same order) and high magnification (**g**, **h**, **i**, in the same order) of the regions highlighted with a red box. Abundant positive (blue) staining for new bone osteoid formation was observed with nano-Si-Ap (**a**, **d**, **g**), with new bone both around and between granules. Formation of new osteoid was observed with nano-SiO_2_-HA (**b**, **e**, **h**) but this was not as developed as nano-Si-Ap. Very limited new bone formation was observed with micro-Si-CaP (**c**, **f**, **i**), with mostly fibrous tissue observed between the granules; red arrow point to a small region of positively stained osteoid. The scale bars are 1 mm, 200 μm and 100 μm

Decalcified histological sections were stained with tetrachrome blue which stains new osteoid tissue a deep blue colour. Sections for the three different materials after implantation for 6 weeks are shown in Fig. 5 at low magnification (a–c) and capture the majority of a typical muscle defect that was filled with 1 cc of bone graft material. The extent of bone formation observed for nano-Si-Ap and nano-SiO_2_-HA across the 4 animals at this early time point was variable and the images shown correspond to the most developed response for each material. At this magnification a clear positive staining of new bone formation, with a trabecular-like structure, can be observed around and between the (decalcified) granules of nano-Si-Ap, Fig. 5a. There was less positive staining in the sections for nano-SiO_2_-HA and an absence for micro-Si-CaP, Fig. 5b and c, with most tissue appearing grey/light blue. Higher magnification images show these results more clearly, with new osteoid tissue appearing on the surface of, and between, the granules of nano-Si-Ap, Fig. 5d and g. For nano-SiO_2_-HA, smaller regions of positively stained osteoid were observed, Fig. 5e and h. For micro-Si-CaP only very small regions of stained osteoid adjacent to some (decalcified) granules (indicated with a red arrow) were occasionally observed with the majority of the tissue surrounding the granules having a fibrous tissue morphology, Figs. 5i.

**Fig. 6:**
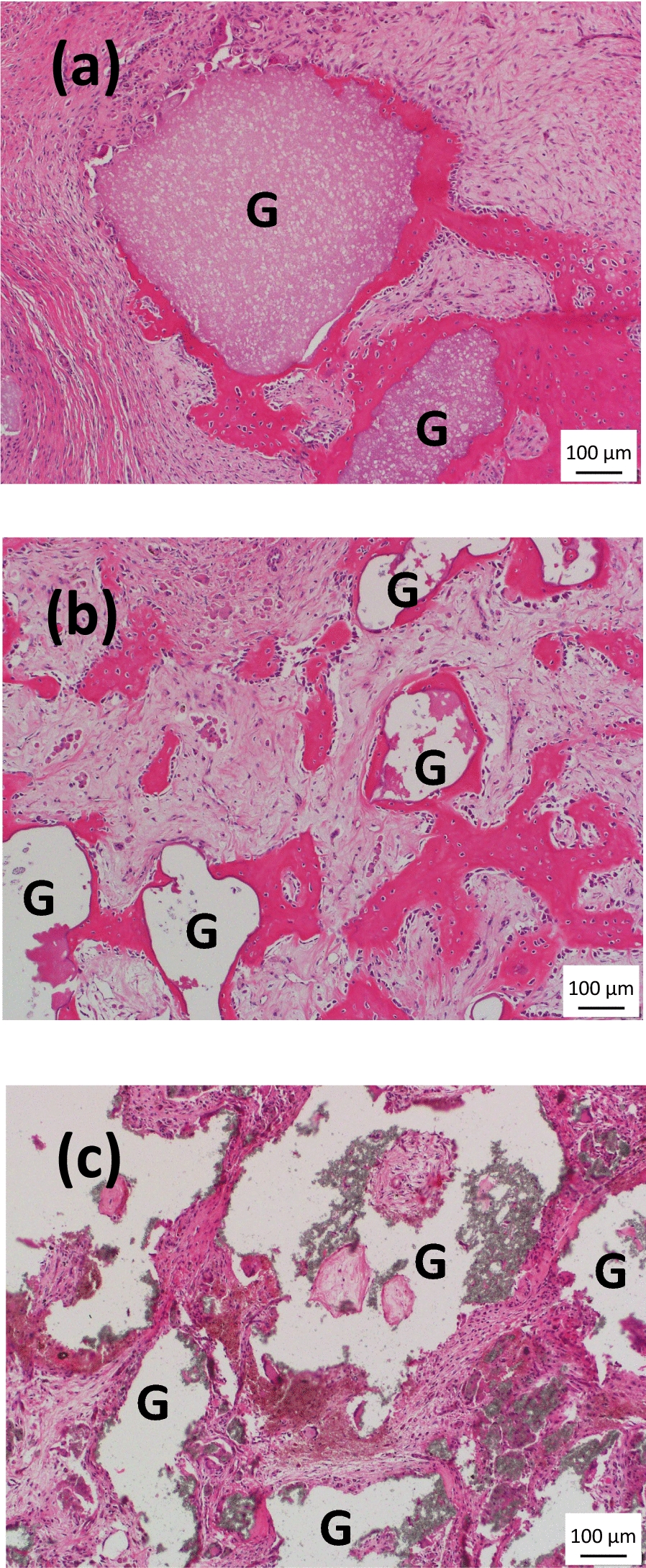
H&E staining of decalcified histological sections from (**a**) nano-Si-Ap, (**b**) nano-SiO_2_-HA and (**c**) micro-Si-CaP after 6 weeks implantation in the paraspinal muscle of sheep (G—Granules). Abundant positive (dark pink) staining for new osteoid formation was observed with nano-Si-Ap (**a**), with new bone both around and between granules, and regions of new osteoid were also observed with nano-SiO_2_-HA (**b**). New osteoid formation was not observed with micro-Si-CaP (**c**). The scale bar is 100 μm

**Fig. 7:**
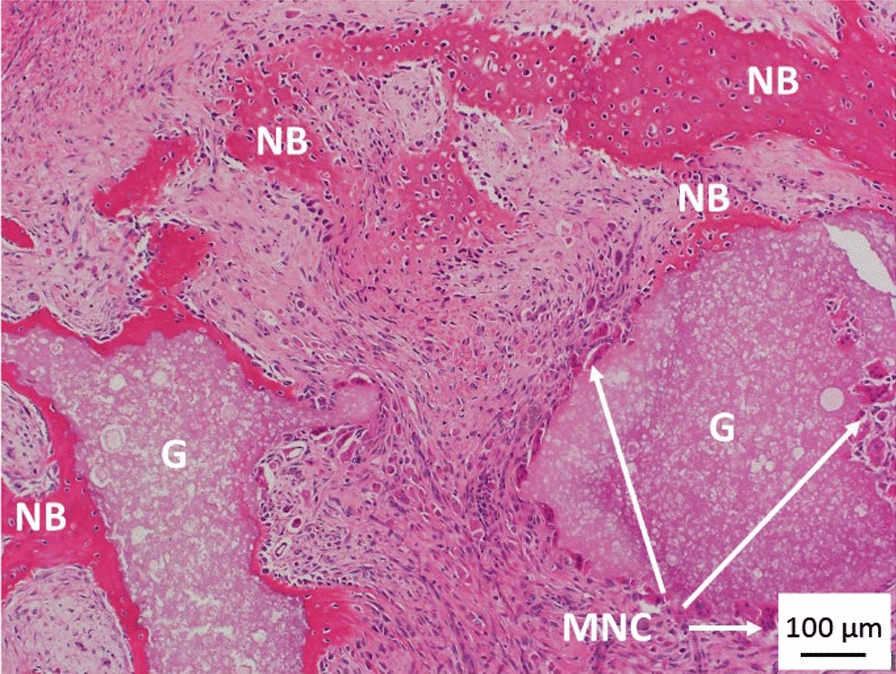
H&E staining of histological sections from nano-Si-Ap after 6 weeks implantation in the paraspinal muscle of sheep. G—Granules, NB—New Bone, MNC – Multi-Nucleated Cells. Abundant positive (red) staining for new osteoid formation, with new bone both around and between granules. Multi-nucleated cells are evident on the surface of some granules (arrows), consistent with cellular remodelling/resorption. Scale bar is 100 μm

These observations were confirmed when decalcified sections were stained with H&E, Fig. 6a, b and c, with positive staining of new osteoid tissue observed as dark pink regions for nano-Si-Ap only, Fig. 6a, again bridging from the surface of one granule to an adjacent granule. Minimal positive staining could be observed for the other two graft materials. In addition to the formation of new osteoid tissue in the nano-Si-Ap materials, some granules were associated with multi-nucleated cells on their surface, and the surface of these granules appeared to have a scalloped or pitted surface, Fig. 7. These observations were not observed with the micro-Si-CaP or nano-SiO_2_-HA.

**Fig. 8:**
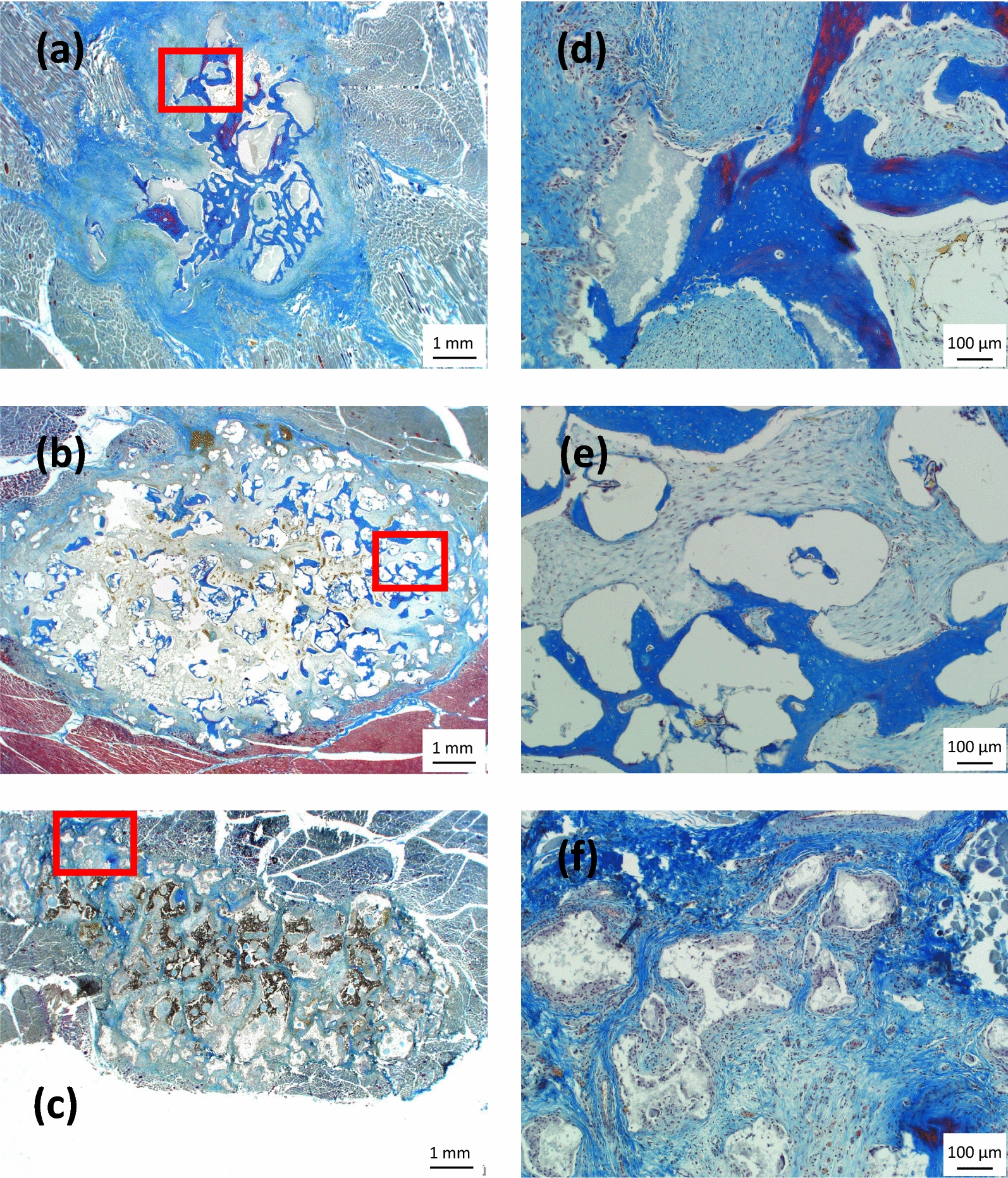
Histological sections with tetrachrome staining of the putty samples implanted in muscle defects for 12 weeks for (**a** and **d**) nano-Si-Ap, (**b** and **e**) nano-SiO_2_-HA and (**c** and **f**) micro-Si-CaP at (**a**, **b**, **c**) low magnification and (**d**, **e**, **f**) high magnification. Scale bars are 1 mm and 100 μm, respectively. Red rectangles indicate the regions expanded to higher magnification

When the materials were implanted in muscle defects for 12 weeks a progression in bone formation was observed with nano-Si-Ap and nano-SiO_2_-HA, but limited change with micro-Si-CaP, Fig. 8. In addition to positive tetrachrome staining of abundant new osteoid in blue, there was also positive staining of mineralised tissue in red, for nano-Si-Ap, Fig. 8a and d, showing a progression in the maturity of the new bone formed. For nano-SiO_2_-HA there was significantly more new osteoid tissue formed compared to at 6 weeks, Fig. 8b, but this was more localised towards the periphery of the defect and was not as abundant as for nano-Si-Ap at either time points. At 12 weeks, osteoid was now bridging between some granules of nano-SiO_2_-HA, Fig. 8e. A small region of the image from micro-Si-CaP showed some positive blue staining of osteoid, Fig. 8f, but this did not appear to show significant progression from the 6-week time point.

**Fig. 9:**
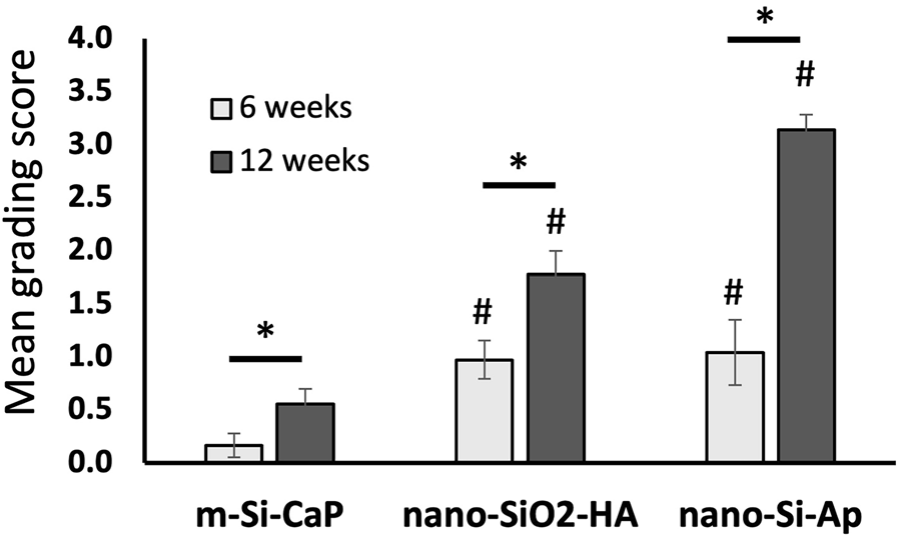
The mean grading scores of bone formation at 6 weeks and 12 weeks for the 3 test groups (mean ± SE). Statistically significant differences between m-Si-CaP and the other two groups were observed at both 6 and 12 week time points (#, *p* < 0.05). All samples showed a significant increase in mean grading score with time (*, *p* < 0.05). Data are the mean values from 3 independent observers grading decalcified histology images from 4 and 2 animals at 6 and 12 weeks, respectively

After blind scoring of the histological images for assessing the amount of bone formation at 6 and 12 weeks, the mean values of the scoring from 0 to 4 were compared between the three groups, Fig. 9. At both time points the micro-Si-CaP resulted in significantly less bone than the nano-Si-Ap or nano-SiO_2_-HA groups. At 6 weeks the mean grading scores for nano-Si-Ap or nano-SiO_2_-HA groups were comparable, and for both groups there was a large and significant increase in the mean grading score after 12 weeks. The mean grading score for nano-Si-Ap at 12 weeks was almost double the mean score for nano-SiO_2_-HA, but the differences were not statistically significant (*p* = 0.094), likely due to the small number of test groups in the study at 12 weeks. This finding is consistent with the histological findings (Fig. 8) where extensive bridging bone has formed throughout the defect in nano-Si-Ap, in contrast to the other two groups, where despite an amount of bone volume being measured, there is little to no intergranular bridging, suggesting an effective physiological repair for the nano-Si-Ap material.

## Discussion

The characterisation of the granules of the three putty samples by XRD and FTIR spectroscopy showed that all three samples were apatite-like phases, with varying levels of crystallite size. There is a limitation of the Scherrer method in determining the crystallite size as it does not provide all dimensions of a non-spherical nano-sized crystallite. The nano-Si-Ap and nano-SiO_2_-HA materials have been described as rod-like [11] or platelet-like particles [18], respectively, but the results shown in Table 1 confirm that these materials are composed of primary particles that can be described as nano-sized. The diffraction pattern of the micro-Si-CaP material consisted of narrow diffraction peaks that is consistent of a material with a primary particle size that is at a micron (μm) scale. Reported SEM analysis of these materials show grains of a mean size scale of 1.02 μm [6].

The morphology of granules in synthetic bone graft substitutes varies considerably in granule size, granule shape, porosity type and porosity size [6]. The granules extracted from the three putty samples in this study varied considerably in their size, shape and porosity, which may all contribute to their bone-healing response [19]. Nanocrystalline calcium phosphates are associated with large values of SSA, with values ranging, for example, from 12 to 50 m^2^/g [20]. The value obtained for the nano-Si-Ap in the present study is consistent with this range but the very large value of SSA for the nano-SiO_2_-HA granules is likely partly associated with the silica gel matrix that is connected with the hydroxyapatite crystals that comprise this material [18].

In this study, the bone forming response was evaluated from intramuscular implantation with three different synthetic calcium phosphate bone grafts, nano-Si-Ap (OssDsign Catalyst), micro-Si-CaP (Inductigraft, or AltaPore in the US) and nano-SiO_2_-HA (Nanobone SBX Putty) at 6 and 12 weeks. We found that at the 6-week time point, the nano-Si-Ap graft material showed significant bone formation and graft resorption when compared with the other two grafts tested.

Significant new bone formation was observed with nano-Si-Ap after 6 weeks compared to the other two graft materials studied here, and qualitatively this was comparable to reported bone formation in some other synthetic bone graft substitutes implanted in intramuscular defects in canines [6, 7] for 12 weeks. Unfortunately, there is limited published data in these large animal intramuscular defect models at time points earlier than 8–12 weeks [21]. By 12 weeks in the present study, nano-Si-Ap exhibited significant resorption/remodelling, observed from histology, X-rays and micro-CT, compared to the 6-week time point and the other grafts tested.

The surface of some granules of nano-Si-Ap that appeared to have MNCs and a scalloped or pitted surface, Fig. 7, may be indicative of cell-mediated resorption, but this would need to be validated further to confirm that this was cell-mediated. The radiographs (Fig. 3) and micro-CT images (Fig. 4a and d) also supported resorption of the nano-Si-Ap graft material from 6 to 12 weeks, with this occurring simultaneously with bone formation. In contrast, the other two graft materials did not show morphological evidence of graft resorption at 6 or 12 weeks. Interestingly, when autograft bone is implanted in intramuscular defects in large animal pre-clinical models, the autograft bone is resorbed over time, after initial new bone formation [22, 23]. For example, autograft was completely resorbed in 50% of the goats used for intramuscular implantation for 12 weeks, and minimal new bone formation was observed [23].

The osteoinductivity of micro-Si-CaP (Inductigraft, or AltaPore in the US) has been reported in sheep intramuscular defects at an end-point of 12 weeks [9], and nano-SiO_2_-HA (Nanobone) was studied in mini-pig intramuscular and subcutaneous defects at end-points of 5 and 10 weeks and 4 and 8 months [8]. The sheep studies with Inductigraft (termed micro-Si-CaP in the current study) showed the presence of some new bone after 12 weeks in high magnification images of the implants, and from these it appears that the new bone was associated with the large macropores in the graft material. The formation of bone tissue associated with Nanobone in mini-pigs was minimal in intramuscular defects at all time points, whereas extensive bone formation was observed in subcutaneous implants from 10 weeks onwards. The large differences observed between the two implantation sites was also reflected in the resorption of the graft material, with minimal resorption from 5 weeks to 8 months for intramuscular implants, but significant graft resorption when implanted subcutaneously. An additional study reported the implantation of Nanobone into muscle defects in goats for 29, 91 or 181 days but no evidence of bone formation was observed at any of the time points, but cell-mediated degradation of the material was confirmed by histology [24].

It is difficult to compare these results with the present study as the Inductigraft study in sheep looked only at a 12-week time point, but the small regions of new bone that we observed with micro-Si-CaP at 12 weeks was also associated with the large macropores. The different species and also the minimal OI response observed intramuscularly but strong response subcutaneously in the Nanobone study in mini-pigs is in contrast to our findings in only intramuscular defects in sheep, but OI has been shown to be species dependant. Although bone formation subcutaneously is an interesting phenomenon and is often used as a test for the OI of demineralised bone matrix (DBM) and biological molecules such as BMP- 2, it is not very clinically relevant to spine and trauma.

This study evaluated three different clinically available synthetic bone graft substitutes, and this does present some limitations in understanding how the chemical and physical properties of the different materials may influence new bone formation and graft resorption. All three materials were calcium phosphate apatites, as shown by XRD analysis, and all three contained silicon, but in two of these the silicon exists as silicate ions substituting for phosphate ions in the apatite structure (micro-Si-CaP and nano-Si-Ap), whereas the other (nano-SiO_2_-HA) it existed as a silica (SiO_2_) matrix surrounding the apatite crystals [18]. The three materials also had different morphologies/microstructures, ranging from nano-sized to micron-sized primary particles, and CaP morphology is known to affect osteoinductivity [25]. It is not possible to directly attribute the effects of these differences in material properties to the in vivo results observed in this study, but it would suggest that the responses observed are a result of multiple properties; data from a small pilot study showed that a silicate-free version of nano-Si-Ap did not result in new bone formation after 6 weeks in the same model as used here [26].

The use of three clinically used bone grafts in this study limited the ability to understand the role that different parameters might have on OI bone formation. A study using a large number of experimental CaP bone grafts with a wide range of physical and chemical properties, that would include the values of the materials studied here, would be needed to isolate the critical parameter or combination of parameters that drives an OI response.

Many studies in the literature have attempted to understand the role of various physical and chemical properties of various CaP bone grafts on in vivo OI. A study of macroporous silicate calcium phosphates showed that the quantity of microporosity in the graft material, typically between 1 and 10 µm in size, affected the OI response in muscle defects in sheep. After 12 weeks significantly more bone was formed in grafts containing 46% microporosity, compared to 22.5% microporosity, with both grafts having the same level of total macroporosity of 80–82.5% [27]. Another study by the same group showed the effect of silicate in the CaP on the level of OI bone formation in a sheep muscle defect after 12 weeks for similar macroporous bone grafts with 80% macroporosity and 30% microporosity; the presence of silicate resulted in significantly more new bone formation than for a non-silicate containing graft [5].

The effect of the specific surface area of porous biphasic CaPs on the formation of bone in a goat intramuscular defect after 12 weeks showed that a high surface area of 1.5–1.8 m^2^/g resulted in bone incidence in 9 out of 11 animals, whereas a lower surface area of 0.2 m^2^/g resulted in no bone formation [28]. The surface area of the micro-Si-CaP in the present study was 0.2 m^2^/g and resulted in negligible bone formation after 12 weeks in the sheep muscle defect, whereas the surface areas of the other two samples studied were much higher than 1.5–1.8 m^2^/g, Table 1.

A comparison of two tricalcium phosphate samples that were produced to have different grain sizes, pore sizes and surface areas, but similar chemistries, showed that the sample with the smaller grain size and pore size, but larger surface area, resulted in bone formation after implantation in a canine intramuscular defect for 12 weeks, whereas no bone formation was observed in the larger grain/pore size and lower surface area sample [29]. While it was unclear from this study if one of these parameters had a significant effect, or if multiple parameters had a synergistic effect on OI bone formation, this binary effect was quite striking.

The effect of the physicochemical properties of six clinically used CaP bone grafts and two experimental TCP bone grafts on OI bone formation after 12 weeks in intramuscular defects in canines was reported [6]. Only three of the eight bone grafts resulted in bone formation after 12 weeks, with only two showing appreciable levels (21.6 and 2.1% bone formed in the available space) compared to the third (0.1% bone formed in the available space). The two samples that formed appreciable levels of bone had different chemistries (100% TCP compared to 55% HA- 45% TCP) but comparable grain sizes, microporosity and surface area; apart from one other material in the study, they had the smallest grain sizes and highest surface areas of the materials studied. The physical parameters of one of the samples in this study (Actifuse) was comparable to the micro-Si-CaP in the present study, although with a lower level of microporosity, while the parameters of another (Bio-Oss) were comparable to the nano-SiO_2_-HA but without the presence of silicate, and they formed 0.1% or 0% bone in the available space after 12 weeks, respectively. The authors were not able to conclude which parameter was critical to inducing bone formation in the muscle defects, or if there were multiple parameters required, even though a wide range of materials were studied.

Findings from the current study showed that osteoinductivity, new bone formation in a non-osseous defect, could be observed after only 6 weeks in a sheep intramuscular defect model, and clear qualitative differences could be observed between the three bone grafts studied at 6 and 12 weeks, including bone formation and graft resorption. The extensive bone formation observed with nano-Si-CaP after only 6 weeks, and progression of bone formation to 12 weeks, may explain why this material resulted in high fusion rates and bone formation at the centre of the graft in a posterolateral fusion study after 6 weeks [11]. In the current environment where it can be challenging for surgeons to assess the relevance of synthetic bone graft claims, even those based on established performance models, such as the Boden model which was developed to evaluate advanced biologics such as rhBMP- 2, there is a need for a model which elucidates not just one outcome at a single timepoint but provides insight into the mechanism of bone repair in order to be able to make more informed clinical decisions based on the outcomes.

The current study has some limitations, and these should be considered if an intramuscular defect model was to be established as a “standard model”. The present study did not include a positive or a negative control, although in previous pilot studies using this model a coral-derived osteoconductive CaP was used effectively as a negative control. Autograft could serve as a suitable positive control for bone formation, but also as a control for cell-mediated graft resorption. The present study was also limited mostly to qualitative assessment of bone formation and graft resorption, and this model can be improved by quantification of these measures over at least two implantation times, and include a time zero for quantification of graft resorption by micro CT and histomorphometry; a minimum of 3–4 animals per time point would be recommended for quantitative analysis. This would provide additional information on bone quality, such as maturity, bone marrow formation and extent of trabecular bridging, present in the defect. Additionally, immunohistochemistry for markers of bone formation and resorption would provide additional insights, although it might be appropriate for a standard model to focus on a small number of accepted markers, compared to studies that aim to study mechanisms of bone formation that would require an extensive panel of markers. As mentioned earlier, a major limitation of previous studies that use a large model of osteoinductivity is the use of a single end-point, usually 12 weeks. The present study supports the use of a much earlier time point, 6 weeks, that can provide granularity in differentiating OI responses of different graft materials, so in addition to including multiple time points, and earlier end-point should be included.

Taken together, such a model would provide an effective companion to preclinical data from a critical sized defect model and a clinically relevant model of posterolateral spinal fusion, providing greater insight into the behaviour of synthetic bone graft substitutes, in particular as these have shown a significant development in sophistication over the last decade.

## Conclusions

The current findings indicate that the implantation of synthetic bone grafts in intramuscular defects in sheep can discriminate the ability to form new bone in this non-osseous site between three clinically available silicate calcium phosphate bone grafts. While these three materials all contain the same poloxamer gel carrier and all contain silicate, they have significant differences in their primary crystallite/particle size, surface area and the form of silicate incorporation into the calcium phosphate granule. The use of two implantation time points provided greater insight into the progression of bone formation and also the identification of any graft resorption/remodelling. Observance of an osteoinductive response from synthetic bone grafts may have a clinical relevance in the treatment of challenging bone defects. The development of a more discriminating standard pre-clinical model for assessing osteoinductivity would potentially better inform the clinical management of challenging cases.

## Supplementary Information

Supplementary material 1: Figure S1 SEM images of the granules removed from the putty samples of (a and d) nano-Si-Ap, (b and e) nano-SiO2-HA and (c and f) micro-Si-CaP at (a, b, c) low magnification and (d, e, f) high magnification. Scale bars are 200 m (a and c), 100 m (b) and 10 m (d–f)

## Data Availability

The datasets used and analyzed in this study are available from the corresponding author on reasonable request.

## Abbreviations

CaP: Calcium phosphate
FTIR: Fourier transform infrared spectroscopy
HA: Hydroxyapatite
H&E: Haematoxylin and eosin
SEM: Scanning electron microscopy
SSA: Specific surface area
TCP: Tricalcium phosphate
XRD: X-ray diffraction
