# Supplementary figures and images for: Early new bone formation in ovine intramuscular defects: comparison between different silicate-containing calcium phosphate synthetic bone grafts
Source: J Orthop Surg Res. 2025 Apr 11;20:369. doi: 10.1186/s13018-025-05740-0 (PMC11987333; doi:10.1186/s13018-025-05740-0)

## Slide 1
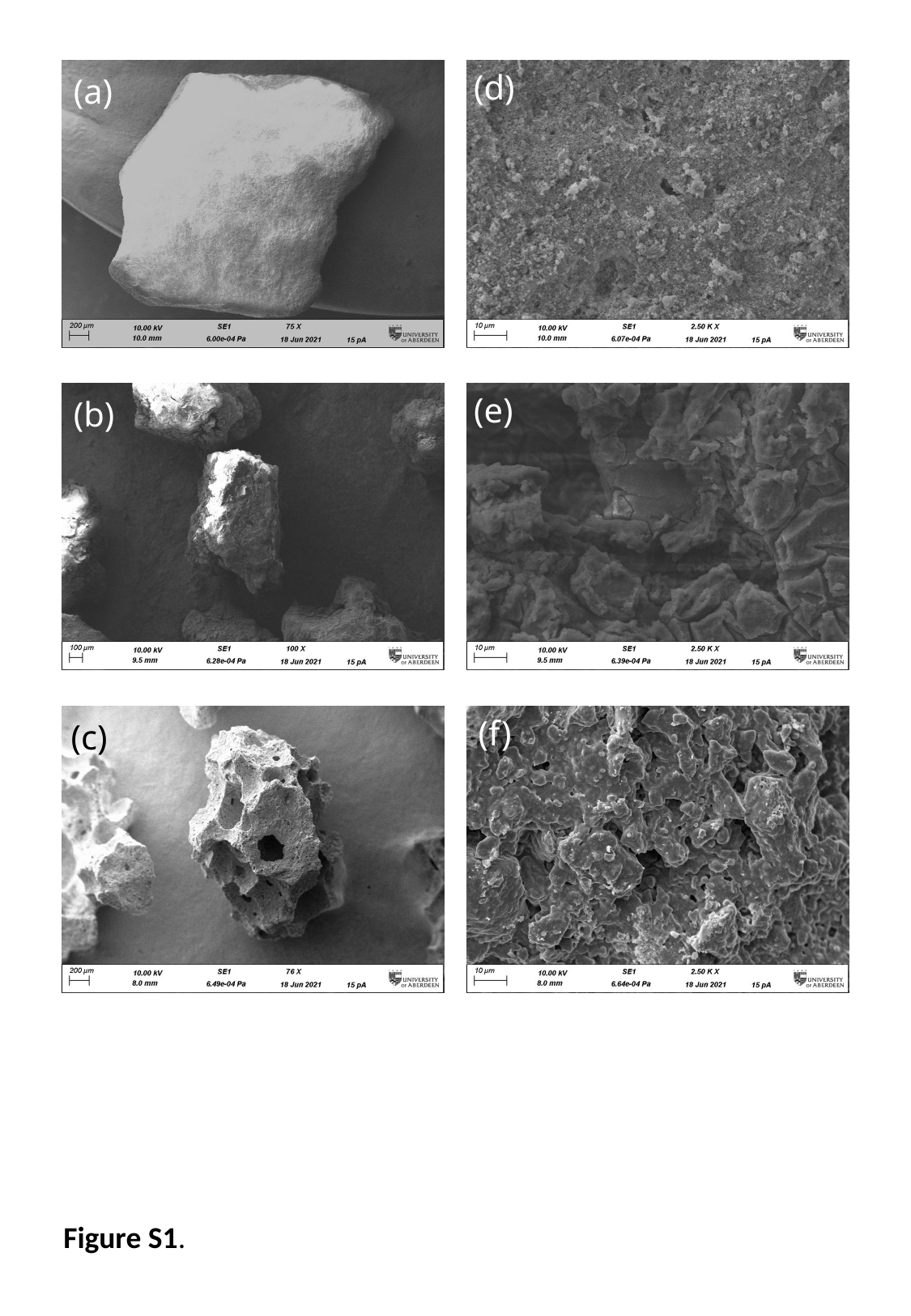

(d)
(a)
(e)
(b)
(f)
(c)
Figure S1.

Supplement: Supplementary file 1 — Supplementary material 1: Figure S1 SEM images of the granules removed from the putty samples of (a and d) nano-Si-Ap, (b and e) nano-SiO2-HA and (c and f) micro-Si-CaP at (a, b, c) low magnification and (d, e, f) high magnification. Scale bars are 200 m (a and c), 100 m (b) and 10 m (d–f) [file 13018_2025_5740_MOESM1_ESM.pptx]
